# Effectiveness and Safety of Posterior Vaginal Repair with Single-Incision, Ultralightweight, Monofilament Propylene Mesh: First Evidence from a Case Series with Short-Term Results

**DOI:** 10.1155/2021/3204145

**Published:** 2021-01-02

**Authors:** Francesco Deltetto, Alessandro Favilli, Giovanni Buzzaccarini, Amerigo Vitagliano

**Affiliations:** ^1^San Luca Nursing Home, Torino, Italy; ^2^Department of Obstetrics and Gynecology, AOUI Verona, Verona, Italy; ^3^Gynecological Division, San Camillo Hospital, Trento, Italy; ^4^Department of Women and Children's Health, Unit of Gynecology and Obstetrics, University of Padua, 35128 Padua, Italy

## Abstract

**Objective:**

The use of transvaginal mesh is controversial, and over time, multiple surgical methods for the treatment of posterior vaginal prolapse (PVP) have been proposed including different surgical approaches and techniques. To date, no clear conclusion has been reached about the use of mesh for reinforcing transvaginal posterior repair. The aim of this study was to evaluate the feasibility, safety, and effectiveness of a novel, ultralightweight mesh for the treatment of PVP.

**Methods:**

We performed a single-center, prospective observational study on consecutive patients referred for primary or recurrent, symptomatic stage II PVP (according to the international Pelvic Organ Prolapse Quantification System) from April 2017 to September 2018. In all patients, transvaginal posterior repair was augmented with a single-incision, isoelastic polypropylene mesh. Data about the postoperative outcomes were collected until December 2019.

**Results:**

A total number of 15 patients were included. The median follow-up after surgery was 18 months (IQR = 14). Surgery was completed in all cases without complications. Regarding the anatomical outcomes (as measured according to POP-q classification), a significant improvement was observed in terms of Bp, D, and C (*p* < 0.05). The functional outcomes were significantly ameliorated after surgery, with a reduction of bulge symptom, stypsis, incomplete evacuation, and excessive staining (*p* < 0.05). The quality of life was significantly improved in the majority of patients (*p* < 0.05). Median patients' satisfaction rate was 100% (IQR = 22.5%). Neither early nor late postoperative complications occurred.

**Conclusions:**

Single-incision, ultralightweight polypropylene meshes were safe and highly effective in the treatment of PVP. As our study has some limitations, further large, controlled studies are needed.

## 1. Introduction

Pelvic organ prolapse (POP) refers to the descent or herniation of one or more pelvic organs from their normal attachment sites or their normal position in the pelvis [1]. POP may variably involve the anterior vaginal wall (cystocele), the posterior vaginal wall (rectocele, enterocele), the uterus (uterine prolapse), and the vaginal apex (apical vaginal prolapse) [1, 2].

POP is a common condition, affecting up to 50% of parous women and being associated with high healthcare costs [3]. At present, the lifetime risk for women undergoing POP surgery is estimated between 6.3% and 19% and is expected to further increase in the near future [4, 5]. For these reasons, the development of effective strategies for treating POP has gained high social priority.

The second most common type of POP is posterior vaginal prolapse (PVP), whose incidence is estimated in 5.7 new cases per 100 women-year [6, 7]. When symptomatic, PVP can be associated with rectal pain and defecatory dysfunctions (such as constipation, tenesmus, splinting, and fecal incontinence), potentially causing a serious deterioration of patients' quality of life [8, 9].

The use of transvaginal mesh is controversial, and over time, multiple surgical methods have been proposed for PVP including different surgical approaches (i.e., transvaginal, transperineal, and transanal) and techniques (i.e., native tissue repair, augmentation with biological graft, or synthetic mesh) [10, 11]. Recent studies were in agreement in demonstrating the superiority of transvaginal repair compared to other surgical approaches for PVP, as the transvaginal approach was associated with the lowest risk of prolapse recurrence [10]. Nevertheless, no clear conclusion has been reached about the use of mesh for reinforcing transvaginal posterior repair (TVP). On the one hand, transvaginal mesh surgery (TVM) may reduce the risk of prolapse recurrence compared to native tissue repair. On the other hand, TVM may be associated with a higher risk of surgical complications including mesh extrusion, perineal pain, and dyspareunia [10, 12]. Therefore, the benefit/risk ratio for TVM is the fundamental point which is currently under scientific scrutiny.

Current evidence on the effectiveness and harms of TVM is mainly limited by the problem that previous studies employed outdated meshes with high weight or transobturator harms, most of whom have been already withdrawn from the market. In recent times, more technological ultralightweight mesh kits have been developed for treating PVP, with some potential advantages compared to the older ones. First, the lighter weight of the meshes may reduce the risk of vaginal extrusion. Moreover, these meshes can be positioned using a minimally invasive technique with a single surgical incision, potentially minimising the surgical trauma and improving patients' postoperative outcomes [13, 14].

Here, we present the first series of patients who undergone TVP with a novel, ultralightweight mesh kit with a median follow-up of 18 months.

## 2. Materials and Methods

### 2.1. Study Design

The present was a single-center case series realized at Gynecological Division, San Camillo Hospital, Trento, Italy. We enrolled consecutive patients referred for PVP in whom TVM was indicated from April 2017 to September 2018. All participants gave written informed consent to use their data for research purposes. The study was exempted from institutional review board (IRB) approval because its design was observational (i.e., without any modification of the routine clinical practice) and all data was anonymized before analysis. All the surgical procedures were performed by a single skilled surgeon (FD) with more than 500 previous TVM surgery procedures. The study was conducted following the IUGA/International Continence Society (ICS) joint report on the terminology for reporting outcomes of surgical procedures for pelvic organ prolapse [15]. Cost analysis was not performed.

### 2.2. Participants

We included patients with a diagnosis of primary or recurrent, symptomatic stage II PVP according to the international Pelvic Organ Prolapse Quantification System (POP-Q) [16]. All patients with rectocele and/or enterocele were eligible. Patients were included exclusively if able to give written informed consent. Exclusion criteria were as follows: malignant diseases, previous TVM surgery, neuromuscular diseases, dementia, and history of chronic pelvic pain.

### 2.3. Mesh Material

**Figure 1:**
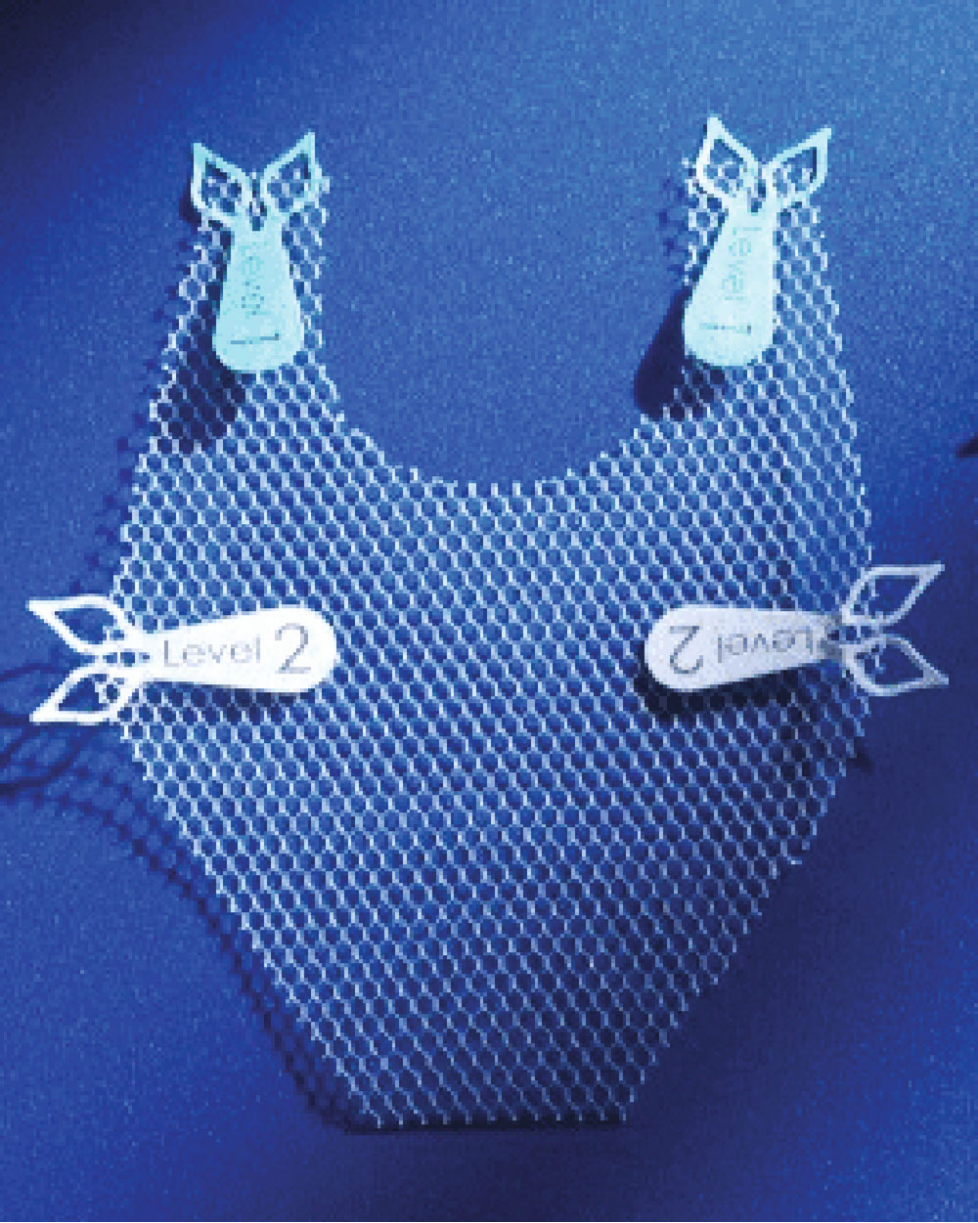
InGYNious (A.M.I., Feldkirch, Austria) ultralightweight, monofilament polypropylene mesh (21 g/m^2^), with a hexagonal structure and six-point suture fixation (three-level support), consisting of extralarge micropores of 100 to 150 *μ*m and macropores with diameters of 1.9 (uniform) to 2.8 mm (maximum).

In all patients, transvaginal posterior repair was augmented with an isoelastic mesh (InGYNious, A.M.I., Feldkirch, Austria). It is ultralightweight, monofilament polypropylene mesh (21 g/m^2^) with a hexagonal structure and six-point suture fixation (three-level support), consisting of extralarge micropores of 100 to 150 *μ*m and macropores with diameters of 1.9 (uniform) to 2.8 mm (maximum) (Figure 1).

### 2.4. Surgical Technique

After infiltration with adrenaline solution into the subfascial posterior vaginal wall, a vertical incision is performed with a cold blade scalpel. The vaginal edges are grabbed with Kocher forceps leaving the fascia adherent to the mucosa.

Now, a delicate digital detachment is performed laterally and downwards until the fibrous nucleus of the perineum. Upwards, lateral detachment slides behind the cervix and the Douglas peritoneum. In the case of conspicuous enterocele, a peritoneum opening may occur and is sutured with a continuous absorbable suture.

The lateral detachment reaches the yellow perirectal fat tissue. Then, the index finger is pushed downwards to the ischial spine. Using the Mayo scissors and the index finger, the bone spike is reached. At this point, the preparation of the sacrospinous ligament is performed by pressing the finger on it. The rectum is then medialized. Slipping down the sacrospinous ligament, the surface of the iliococcygeus muscle is easily identified for the reconstitution of the II level of De Lancey.

At this point, laterally to the fibrous nucleus of the perineum, the deep perineal transverse muscles are found for the reconstruction of the III level.

**Figure 2:**
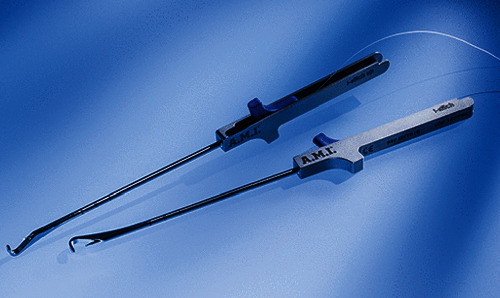
I-stitch. Reusable instrument for fixation of suture material to the tissue.

With the *i-Stitch* device (A.M.I., Feldkirch, Austria; Figure 2), the sacrospinous ligament and the ileococcygeal muscle are reached to put polypropylene sutures and these sutures pass through the mesh too. One end of the sutures fixed to the ligament sacrospinous goes through the cervix. Then, the distal mesh is connected to the deep transverse perineum muscle. Now, the sutures are tied. All the steps are bilateral. At the end, the vaginal suture is performed.

Repairing a posterior defect after hysterectomy and then without the cervix, the mesh does not have to hook to the vagina in order to reduce extrusion risk. Another absorbable suture is passed through the sacrospinous ligaments to fix up and suspend the vagina. They are tied after the vaginal closure as the final step of the intervention.

### 2.5. Data Collection and Follow-Up

Two authors with adequate training in pelvic surgery (F.D. and A.V.) completed the data collection prospectively. Data included patients' general features (i.e., age, BMI, and menopausal status), indication to surgery (including the type of POP, POP-related symptoms, quality of life, and sexual activity), perioperative data (i.e., duration of the intervention, blood loss, perioperative complications, length of hospitalization, and need for analgesia), and postoperative data (objective anatomical outcomes, functional outcomes, sexual life after surgery, degree of satisfaction about the VPM, quality of life, and postoperative complications). The sexual function after surgery was assessed by using a validated questionnaire (Pelvic Organ Prolapse/Urinary Incontinence Sexual Questionnaire (PISQ-12)) [17].

The evaluation timeline was very early (1 and 3 months after surgery), early (every 3 months until the completion of the first year after surgery), and intermediate (every 3 months after the first year after surgery). Information was collected clinically (by gynecological check-up) and by periodic telephonic interviews (every 6 months) until December 2019. All the data were inserted in an electronic database in Excel format.

### 2.6. Statistical Analysis

Statistical analysis was performed using the Statistical Package for Social Sciences (SPSS) v. 22.0 (IBM Inc., Armonk, NY, USA). Continuous variables were reported as median with interquartile range (IQR). Qualitative variables were presented as frequencies (absolute) and percentages (%). Differences between preoperative and postoperative evaluations were analyzed by using the Wilcoxon signed-rank test for matched pairs for numerical variables and the McNemar test for categorical variables. The level of significance was set at *p* = 0.05. Power analysis was not feasible.

## 3. Results

**Figure 3:**
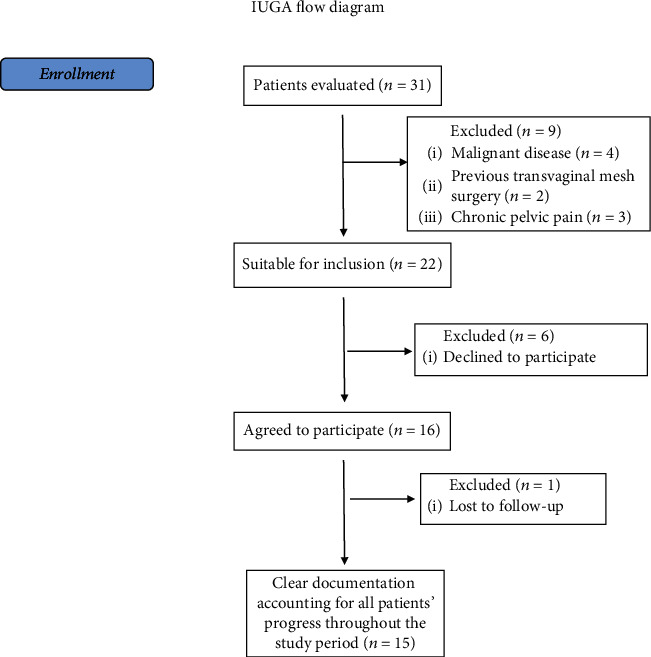
IUGA flow-diagram of patients' enrollment.

We assessed for eligibility a total number of 31 patients. 15 patients were finally included after applying our inclusion and exclusion criteria (Figure 3). The median age at the time of surgery was 69 years (IQR = 14), and the median BMI was 25 (IQR = 7). Nine patients had a history of pelvic prolapse surgery (*n* = 4 vaginal hysterectomy, *n* = 3 vaginal hysterectomy plus posterior repair, and *n* = 2 cystocele repair). The indication for TVM was symptomatic enterorectocele in 10 cases (66.7%), rectocele in four cases (36.3%), and isolated enterocele in a single patient (6.7%). The most common symptoms were bulge (*n* = 15, 100%), incomplete evacuation (*n* = 11, 73.3%), stypsis (*n* = 10, 66.7%), excessive staining (*n* = 10, 66.7%), and urgency (*n* = 10, 66.7%) (Table 1).

The median duration of the surgical procedure was 50 minutes (IQR = 20), and the median blood loss was 20 cc (IQR = 10). The median hospital stay was 2 days (IQR = 1), and the median time to return to daily activities was 18 days (IQR = 8). The median follow-up after surgery was 18 months (IQR = 14). In a single case, the follow-up was interrupted before the end of the study (i.e., 15 months after surgery) due to cerebral hemorrhage and sudden death. No perioperative complications were recorded.

Regarding the anatomical outcomes (as measured according to POP-q classification at the last follow-up visit compared to preoperative evaluation), a significant improvement was observed in terms of Bp (-2 (IQR = 1) vs. 2 (IQR = 1), *p* = 0.001), Ap (-2 (IQR = 1) vs. 2 (IQR = 2), *p* = 0.001), D (-3 (IQR = 4) vs. 2 (IQR = 3), *p* = 0.003), C (-4 (IQR = 1) vs. 0 (IQR = 6), *p* = 0.005) with no differences in Ba (-2 (IQR = 1) vs. -2 (IQR = 1), *p* = ns), and Aa (-2 (IQR = 1) vs. -2 (IQR = 1), *p* = ns). Additionally, the QoL was significantly improved in the majority of patients (i.e., ameliorated in nine patients, unchanged in three patients, worsened in one patient, and not estimable in a single case (*p* = 0.03)).

The functional outcomes were considerably ameliorated after surgery, with a significant reduction of bulge symptom (20% vs. 100%, *p* = 0.001), stypsis (13% vs. 66.7%, *p* = 0.01), incomplete evacuation (13% vs. 73.3%, *p* = 0.007), and excessive staining (13% vs. 66.7%, *p* = 0.01). Differently, a moderate but nonstatistically significant amelioration in the urge symptom (33.3% vs. 66.7%, *p* = ns) and digital evacuation (0% vs. 13.3%) was recorded.

Only seven out of 15 reported an active sexual life before surgery, of whom three experienced an improvement in sexual activity and four did not report any change after surgery (*p* = ns). In the remaining patients, the sexual activity before and after surgery was not measurable. No patient experienced postoperative dyspareunia.

**Figure 4:**
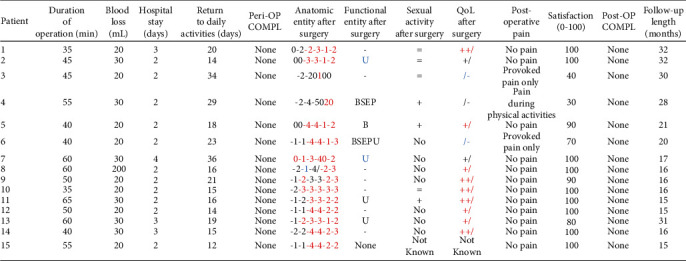
Perioperative data. Peri-OP COMPL: perioperative complications. Anatomic entity: the number are representative of POP-Q anatomic classification (Pelvic Organ Prolapse Quantification) following the International Continence Society (ICS). The numbers are in centimeter, in order: Aa (anterior vaginal wall caudally), Ba (anterior vaginal wall cranially), C (more declive vaginal point), D (posterior fornix), Ap (posterior vaginal wall caudally), and Bp (posterior vaginal wall cranially). Positive numbers mean a more prolapsed structure; negative numbers mean a more cranially structure. In red, we highlighted the improvement in anatomic defects; in blue, the worsening. Functional entity: S: stypsis; D: digital use; E: incomplete evacuation; P: excessive straining (all considered for symptoms more frequently than 1/month); B: vaginal bulge (b for few symptoms); U: urgency. As above, in blue, the worsening of symptoms. QoL: quality of life scale; ++/: excellent; +/: good; +/-: tolerated; /-: poor; /--: terrible. In red, we highlighted the improvement in quality of life score; in blue, the worsening. Post-OP COMPL: postoperative complications.

Importantly, the majority of patients were considerably satisfied with their postoperative outcomes, with a median satisfaction rate of 100% (IQR = 22.5%). Finally, neither early nor late postoperative complications occurred. All data about perioperative and postoperative outcomes are reported in Figure 4.

## 4. Discussion

### 4.1. Main Findings

To the best of our knowledge, this is the first study investigating the feasibility, safety, and effectiveness of an ultralightweight mesh (*InGYNious*) for PVP repair. Importantly, the median duration of surgery was 50 minutes and did not exceed 65 minutes in any case, suggesting a fast and reproducible surgical technique. In particular, when comparing our data with those from other studies on laparoscopic PVP mesh repair, the time needed for surgery was considerably lower in our experience (mean time of laparoscopic POP repair > 100 minutes) [10, 18]. Additionally, the use of spinal anaesthesia and no requirement of Trendelenburg position represent considerable advantages of our technique for surgery in elderly women.

Moreover, our experience substantiates the safety of this novel surgical approach, as the procedures were completed with minimal blood loss (median = 20 cc) and no perioperative complications. These findings are inconsistent with those from previous studies on vaginal PVP repair, in whom mesh augmentation was associated with a high risk of surgical complications [10, 18–21]. In this respect, we may speculate that our positive results are due to the use of a single-incision, simplified technique, with minimal surgical invasivity and a lower risk of complications.

Notably, we found a significant improvement in both anatomical (i.e., Bp, Ap, D, and C as measured with POP-q classification) and functional aspects after surgery (i.e., bulge symptom, stypsis, incomplete evacuation, and excessive staining), resulting in improved QoL and high patients' satisfaction rate. We believe these two latter points to be of crucial importance, as the primary aim of POP surgery is the improvement of the physical, psychological, and social wellbeing of patients, rather than the mere resolution of anatomic defects.

Additionally, after a median follow-up of 18 months, we did not record any mesh-related surgical complication, including not even a single case of vaginal extrusion. These data may reassure both physicians and patients about the safety of vaginal augmentation with novel, macroporous, ultralightweight polypropylene meshes. For the sake of clarity, we need to stress that current evidence on the safety of TVM is mainly based on trials that are not exempted from limitations. The main issue is the high variability within studies in terms of mesh characteristics and surgical techniques for vaginal mesh augmentation [22]. Such a methodological heterogeneity may inevitably expose to variability in the intervention effects and, therefore, to bias in pooled effect estimates. For instance, we may speculate that ultraweight macroporous meshes, as positioned under the pubocervical fascia, may reduce the risk of vaginal extrusion. Nevertheless, our hypotheses need future confirmation by well-conducted trials with homogeneous interventions.

### 4.2. Strengths and Limitations

The key strengths of this study are the long follow-up, the use of a single surgical technique (including a single mesh type), and the involvement of a single surgeon with high expertise in POP surgery. The main weaknesses are inherent to study design (i.e., lack of a control group) and small sample size, potentially limiting drawing firm conclusions from the data.

## 5. Conclusions

Single-incision, ultralightweight polypropylene meshes were safe and highly effective in the treatment of PVP. TVM was associated with significant improvement in the anatomical and functional outcomes, better QoL, and high patients' satisfaction rate. Moreover, the surgical time was limited and no perioperative and postoperative complications occurred. Accordingly, TVM with ultralightweight polypropylene meshes may be considered as a promising option for PVP repair. As our study has some limitations, further large, controlled studies with long-term follow-up are needed to confirm our findings.

## Figures and Tables

**Table 1:** Preoperative data.

Patient	Age at surgery	Parity	BMI at surgery	Menopause status	HRT usage	Smoking	Chronic cough	Previous Gyn surgery	Defects	Anatomic entity	Functional entity	Sexual activity	QoL
1	80	4	21	Yes	No	No	No	No	ER	0-24432	BSEPU	No	/--
2	57	2	28	Yes	Yes	No	No	HP	ER	003301	BU	Yes	+/
3	69	3	24	Yes	No	No	No	H	E	-2-20300	B	No	+/
4	64	2	33	Yes	Yes	Yes	Yes	No	R	-2-4-5041	BSEP	Yes	/-
5	71	3	26	Yes	Yes	No	No	HP	ER	00-1113	BSEP	Yes	+/-
6	78	1	27	Yes	No	No	No	No	ER	-1-10213	BSEPU	No	+/-
7	93	2	25	Yes	No	Yes	No	No	ER	226736	BSEPU	No	+/
8	60	3	34	Yes	Yes	No	No	No	R	-2-2-4-422	BSEDU	No	+/-
9	76	2	32	Yes	No	No	No	No	R	-1-1-3-332	BSEDP	No	+/-
10	68	2	18	Yes	No	Yes	No	C	ER	-2-2-3333	BPU	Yes	+/-
11	55	0	24	No	-	Yes	Yes	H	ER	-1-24424	BSEPU	Yes	+/
12	67	2	21	Yes	No	No	No	C	ER	-1-11122	BSEPU	No	/--
13	83	5	21	Yes	No	No	No	HP	ER	-1-12312	BSEPU	No	/--
14	72	1	25	Yes	Yes	No	No	H	ER	-2-2-1222	BEU	No	+/-
15	69	2	21	Yes	No	No	No	H	R	-2-2-2-313	B	No	+/

Legend: HRT: hormone replacement therapy. Previous Gyn surgery: H: hysterectomy; P: prolapse correction; C: cystocele correction. Defects: E: enterocele; R: rectocele; A: anterior wall prolapse. Anatomic entity: the number is representative of POP-Q anatomic classification (Pelvic Organ Prolapse Quantification) following the International Continence Society (ICS). The numbers are in centimeter, in order: Aa (anterior vaginal wall caudally), Ba (anterior vaginal wall cranially), C (more declive vaginal point), D (posterior fornix), Ap (posterior vaginal wall caudally), and Bp (posterior vaginal wall cranially). Positive numbers mean a more prolapsed structure; negative numbers mean a more cranially structure. Functional entity: S: stypsis; D: digital use; E: incomplete evacuation; P: excessive straining (all considered for symptoms more frequently than 1/month); B: vaginal bulge (b for few symptoms); U: urgency. QoL: quality of life scale; ++/: excellent; +/: food; +/-: tolerated; /-: poor; /--: terrible.

## Data Availability

We declare that all data are available within the study tables.
